# Fluorosurfactants‐Directed Preparation of Homogeneous and Hierarchical‐Porosity CMP Aerogels for Gas Sorption and Oil Cleanup

**DOI:** 10.1002/advs.201400006

**Published:** 2015-01-28

**Authors:** Ran Du, Zhe Zheng, Nannan Mao, Na Zhang, Wenping Hu, Jin Zhang

**Affiliations:** ^1^ Center for NanochemistryBeijing National Laboratory for Molecular SciencesKey Laboratory for the Physics and Chemistry of NanodevicesState Key Laboratory for Structural Chemistry of Unstable and Stable SpeciesCollege of Chemistry and Molecular EngineeringPeking UniversityBeijing100871P.R. China; ^2^ Collaborative Innovation Center of Chemical Science and Engineering (Tianjin)Department of ChemistrySchool of ScienceTianjin UniversityTianjin300072P.R.China

**Keywords:** Conjugated microporous polymers, aerogel, homogeneous, fluorosurfactant, sorption

## Abstract

**Homogeneous, hierarchical‐porosity and highly hydrophobic conjugated microporous polymer (CMP) aerogels** are facilely prepared assisted by fluorosurfactants. The fluorosurfactants show several roles in controlling the gelation process, modulating pore structures, and raising the hydrophobicity of materials, thus giving rise to aerogels with exceptional gas sorption and oil cleanup performance.

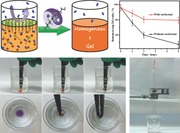

Aerogels are a kind of monolithic three‐dimensional (3D) porous solid network featuring a variety of appealing properties. Hence they have received broad attention from multi‐disciplined researchers and are widely used in many fields.^1^, ^2^, ^3^ Aerogels are normally prepared from the sol–gel chemistry and followed special drying process (e.g., supercritical drying or freeze drying).^2^ Apart from traditional aerogels based on silica, carbon, and some transition metal oxides,^3^, ^4^, ^5^ many new members such as carbon nanotubes (CNT), graphene and metal‐based aerogels with enhanced performance and enlarged application territory have been developed.^6^, ^7^ In the past few years, conjugated microporous polymers (CMPs), combining both dominant microporosity and fully conjugated structure, have attracted tremendous attention and have shown promising applications in various fields.^8^, ^9^, ^10^, ^11^ Recently, we have reported the synthesis of conjugated microporous polymers (CMPs) aerogels, i.e., poly(1, 3, 5‐triethynylbenzene) (PTEB) aerogels in air by the polymerization of 1,3,5‐triethynylbenzene (TEB) and followed freeze‐drying process.^12^ Enjoying features of both CMPs and aerogels, a new type aerogels showed remarkable sorption performance outperforming most CMP‐based materials have been created.

To date, for almost all aerogels, the emphasis has been placed on the chemical composition,^13^ solvent,^14^, ^15^ and some general reaction conditions (temperature, time, and pressure).^16^ The gel with homogeneous chemical composition can be easily obtained without considering environmental atmosphere in the reaction process. The case is totally different in the PTEB gel system, where the ambient atmosphere plays a vital role in the gelation process. The PTEB gel was prepared by the Glaser coupling reaction (Hay's condition^17^ between acetylenic bonds of TEB monomers. According to the synthetic mechanism, the oxygen is involved in the reaction as one of indispensable ingredients.^18^ Therefore, in this type of oxygen‐required reaction, especially for undisturbed condition for gel formation, maintaining a uniform dissolved oxygen (DO) distribution in the precursor solution is crucial for obtaining homogeneous gels. Few studies have concerned the development of hierarchical porous CMPs for effective mass transfer. With dominant micropores, the oil cleanup ability of most CMP‐based materials is far from satisfactory. Moreover, to acquire more efficient oil sorption performance, especially for use in an oil/water mixture, further improvement of the hydrophobicity is required. Hence to handle this new type of environmental‐atmosphere‐sensitive gelation process, to efficiently modulate the pore‐size distribution and gas sorption properties, and to gain a higher hydrophobicity for selective oil sorption, a brand new gelation strategy that is completely different from traditional routes is required.

The key point to acquiring the homogeneous gel is to realize a uniform DO distribution in a precursor solution throughout the whole reaction period. The dominant microporosity of CMP aerogels stems from 3D cross‐linked networks that might be controlled by altering the polymerization degree,^19^ while improvement of hydrophobicity can be realized by introducing a highly hydrophobic component, such as fluorine‐containing substance. In this regard, we propose a fluorosurfactant‐assisted gelation method to alter the reaction path of Glaser coupling and the chemical composition of final materials (shown in **Figure** **1**a), and thereby providing a feasible way to solve problems mentioned above. The fluorosurfactant can play several roles in preparation of CMP aerogels. i) The fluorosurfactant can adsorb on the gas–liquid surface and block the penetration of oxygen from air into solution during reaction process, thus realizing a uniform DO distribution and resultant homogeneous gels. ii) The fluorosurfactant has nitrogen and oxygen atoms with lone electron pairs, which are capable of reducing polymerization degree and altering final pore structures by coordinating the catalyst Cu(I).^20^ iii) The highly hydrophobicity of fluorinated alkyl chain can increase the hydrophobicity of materials,^21^, ^22^ triggering a more efficient oil uptake process. Based on the above analysis, we developed a brand‐new strategy, i.e., the fluorosurfactant‐assisted Glaser coupling reaction to obtain homogeneous and highly hydrophobic CMP‐based aerogels. In this manner, the homogeneous gelation involving an environmental‐atmosphere‐sensitive reaction is realized. More importantly, fluorosurfactants can serve as a unique additive to efficiently modulate the morphology, porosity and gas sorption properties of aerogels. As a result, the aerogel shows not only excellent and tunable gas sorption capacity (0.41–3.47 mmol g^−1^ for CO_2_ and 0.12–0.95 mmol g^−1^ for CH_4_) comparable to conventional CMP materials, but also exceptional oil cleanup performance (20–48 times weight gain) among all MOPs to date.

**Figure 1:**
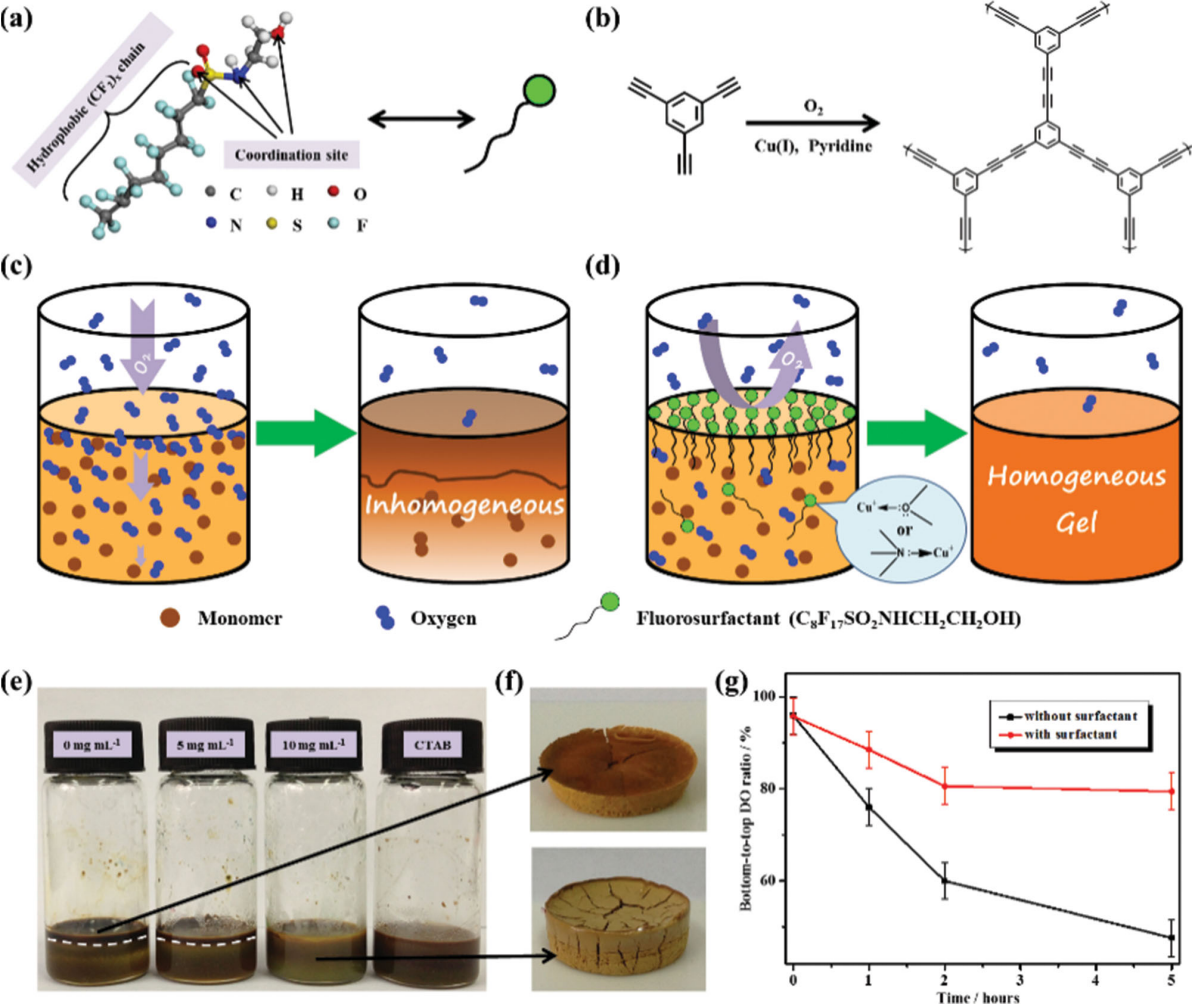
Illustration of synthetic strategy for homogeneous CMP aerogels by a fluorosurfactant‐assisted Glaser coupling reaction. a) The molecular structure and the functions of the employed fluorosurfactant. b) The reaction condition of Glaser coupling reaction (Hay's condition). c,d) Schematic diagrams of the gelation mechanism for PTEB gels without and with fluorosurfactants, respectively. e) PTEB wet gel with different initial fluorosurfactant concentrations (three samples on left) and with 10 mg mL^−1^ CTAB. The white dashed line indicates the interface between top dark gel layer and bottom light gel. f) showed the corresponding aerogels after freeze‐drying. g) The in situ ratio of bottom‐to‐top dissolved oxygen (DO) variation with time evolution.

Briefly, the catalyst (CuCl) and a certain amount of fluorosurfactant were mixed together in pyridine, followed by addition of the pyridine solution of TEB. The mixture was placed at 40 °C water bath for three days to allow the gel formation before purification process. After drying, the PTEB‐based aerogel with the density ranging from 30–60 mg cm^−3^ was obtained. The successful formation of PTEB can be evidenced from the appeared peak at 2221 cm^−1^ in Raman spectra.^12^, ^23^ The resultant aerogels without or with initial fluorosurfactants concentration of x mg mL^−1^ were denoted as PTEB‐0 and PTEB‐F‐x, respectively.

From Figure 1e, without enough fluorosurfactant, the resultant gel was inhomogeneous and showed an obvious gradient distribution along the height direction. A clear line approaching the air‐solvent interface can be seen. Above the line was a dark brown gel layer, while the color of gel below the line was much lighter. For the sample without surfactant, the bottom section even remained in a liquid state, suggesting a very low reaction degree. By contrast, sufficient fluorosurfactant results in a homogeneous brownish yellow gel. When fluorosurfactant was replaced by another non‐fluorosurfactant (hexadecyl trimethyl ammonium bromide, CTAB), the gel was uniform while the color was dark brown, which may mean that CTAB has little effect on the polymerization degree. After purification and drying, the homogeneity of resultant aerogels showed the same trend with wet gels. Owing to the merit of fluorosurfactant, the inhomogeneity problem was solved and thus the scale‐up production could be realized (Figure 1e–f, Figure S2, Supporting Information).

To gain insight into the fluorosurfactant‐assisted homogeneous gelation, analysis should be started from the fundamental coupling mechanism. From a widely accepted Glaser coupling (Hay's condition) mechanism, oxygen was added into the reaction as an indispensable gradient, which was capable of forming an intermediate four‐ring complex, according to DFT calculations.^18^ Previously, we have also shown that nitrogen saturation would block the coupling reaction in this condition.^12^ Based on this mechanism, the reaction can readily proceed in an air atmosphere.

In the natural state, the dissolved oxygen (DO) in the solvent (<1 vol%) was much lower than oxygen concentration in the air (ca. 20 vol%), far from the requirement for complete reaction (Figure S3, Supporting Information). It can be expected that the oxygen in the air will supply the solution to compensate oxygen consumption during the coupling reaction. However, during the process of penetrating into the solution, oxygen molecules were continuously consumed by reacting with monomers, hence few oxygen molecules can finally reach the bottom. Therefore, the DO value exhibited a gradient distribution along the height direction from the gas‐liquid interface (higher DO) to bulk phase (lower DO) as shown in Figure S3, Supporting Information. On the other hand, as the highest DO value was acquired at the air‐solvent interface, the interface solution would undergo a fast reaction and form a dense gel layer at the top section. This layer might block the following oxygen penetration and thus further enlarge the bottom‐to‐up reaction degree difference. Hence it was understandable that once the reaction system was scaled, the low oxygen diffusion efficiency will give rise to the inhomogeneous gel as shown in Figure 1c.

Fluorosurfactants are much more stable and surface active compared to their hydrocarbon analogs, facilitating their use in many rigorous conditions.^21^, ^22^ Surfactants tend to migrate to and orientate at a two‐phase interface owing to their amphiphilic structure.^22^ Above a critical micelle concentration known as CMC, the surfactants will cover the whole air‐liquid interface and micelles appear. Hence, it is expected that when enough surfactants were involved, the air‐solvent interface would be “sealed” and avoid further oxygen penetration. In this way, after fully mixing at the initial stage, the uniform DO distribution would not be considerably affected by the oxygen from air phase, which allowed a homogeneous gelation process by addition of either fluorosurfactant or CTAB as shown in Figure 1d.

However, the used fluorosurfactants contains nitrogen and oxygen atoms that have lone pair electrons, capable of coordinating catalyst Cu(I) ions.^18^, ^20^ The coordination of Cu(I) ions can be easily evidenced from the color evolution upon the addition of fluorosurfactant. Interestingly, from the coupling mechanism, the Cu(I) ions is coordinated by amine at first, enabling further reaction with acetylene.^18^ It is known that small amines such as *N*,*N*,*N*′,*N*′‐tetraethylenediamine (TMEDA) and pyridine can provide lone pair electrons, and thus forming complexion with Cu(I) and facilitating reaction.^12^, ^17^, ^18^ However, as a kind of “large” amine, the fluorosurfactant can not only coordinate Cu(I) ions by nitrogen or oxygen atom with a lone electron pair, but also screen the contact of coordinated Cu(I) with the monomer by its long fluorinated alkyl tail. Therefore, the fluorosurfactants can act as a catalyst‐storehouse and control the free Cu(I) ions at a low concentration level. With reaction proceeding, Cu(I) ions can be slowly released by fluorosurfactant resulted from the complexation equilibrium, and then re‐coordinated with pyridine to trigger subsequent reaction. Hence the reaction rate was greatly slow down, allowing more sufficient species diffusion and thus more homogeneous gelation process.^25^ Because most catalysts were captured by the surfactant coordination, the resultant CMPs gel showed lower polymerization degree than that of surfactant‐free, which can be evidenced by a light color (Figure 1e–f). It was notable that too much surfactant would retard the gelation because of an extremely low catalysts concentration (Figure S2, Supporting Information). By comparison, as CTAB does not have hetero atoms with lone electron pairs, it was reasonable that the concentration of Cu(I) catalyst remained unchanged and the color of the resultant gel was dark brown (Figure 1e), similar to that of surfactant‐free one.

Except for macroscopic observation such as the material color, the above analysis was also supported by in situ dissolved oxygen measurement as shown in Figure 1g, Figure S3, Supporting Information. The DO was in situ measured during the reaction process, and the DO values at different height were recorded. It was found that addition of fluorosurfactants can significantly suppress the oxygen consumption at all inspection time scale, suggesting a significantly decreased reaction rate. By comparing the DO value at the bottom section and the top section of reaction mixture, we can clearly see that the addition of fluorosurfactant considerably reduced the DO difference along the height direction, which was of paramount importance for homogeneous gelation. In principle, this surfactant‐assisted method can also be applied into other atmosphere‐dependent gelation process, which was of great importance for scaling production of many kinds of gels.

The effect of fluorosurfactants on polymerization degree reduction can not only be deduced from a lighter color, but also supported by infrared (IR) spectra of PTEB aerogel with different initial surfactants concentration (**Figure** **2**a). The I_3301_(acetylenic C–H stretching vibration)/I_1575_(aromatic C=C stretching vibration) and the I_2116_(terminal C≡C stretching vibration)/I_2210_(C≡C stretching vibration in phenyl diacetylene) increased with the increasing surfactants amount, suggesting the fluorosurfactants can suppress the polymerization and result in a lower polymerization degree.

**Figure 2:**
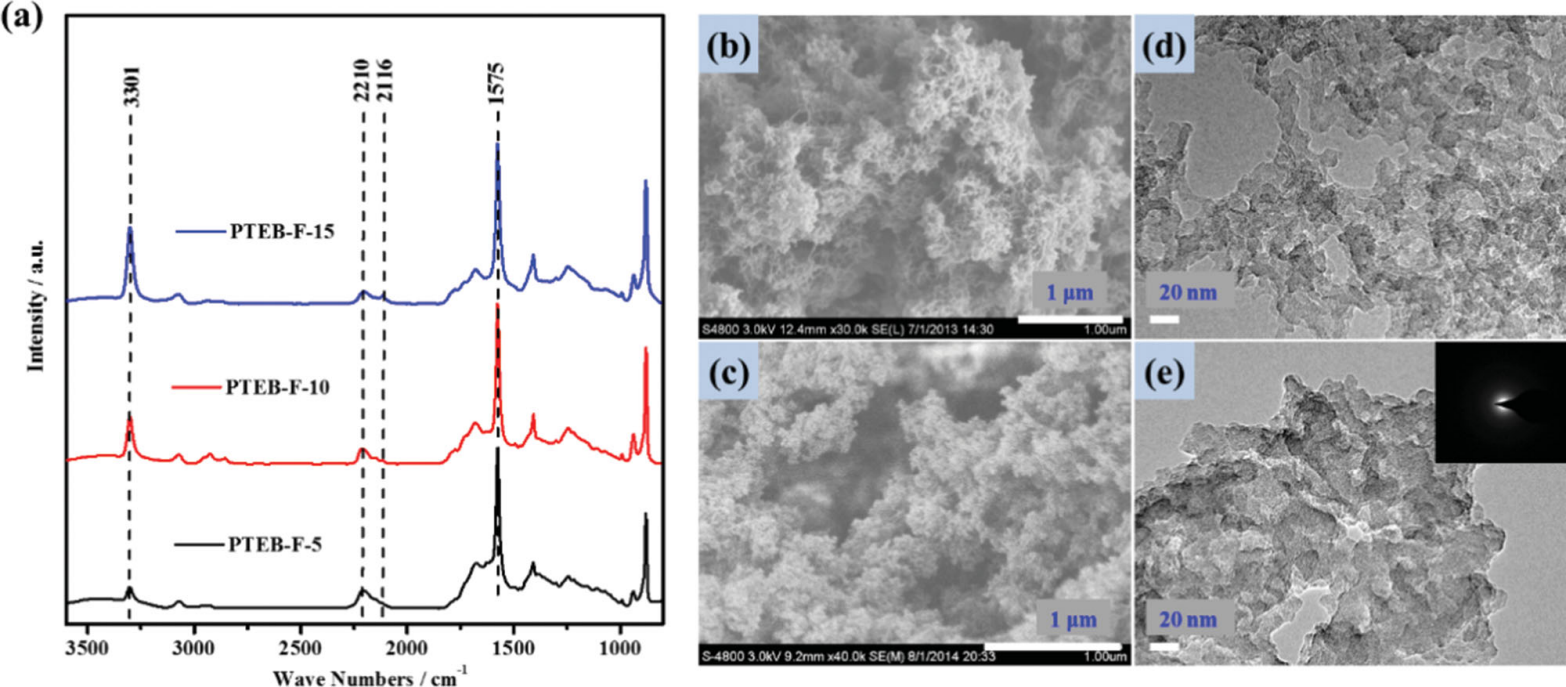
Spectra and morphology characterization. a) The superposed IR spectra of PTEB aerogels with different initial fluorosurfactant concentration (5, 10, and 15mg mL^−1^). b,c) SEM images of freeze‐dried PTEB‐0 and PTEB‐F‐10, respectively. d,e) TEM images of freeze‐dried PTEB‐0 and PTEB‐F‐10, respectively. The inset shows the SAED pattern of the corresponding aerogel.

The morphology of aerogels can also be modulated by fluorosurfactants. From scanning electron microscopy (SEM) (Figure 2b–c, Figure S4, Supporting Information) images, the surfactant‐free aerogel was composed of short fiber‐like building blocks, while the PTEB‐F‐10 was made up of nanoparticles. This might also be attributed to the low polymerization degree of the latter sample. When take a closer look, transmission electron microscopy (TEM) images (Figure 2d–e, Figure S5, Supporting Information) showed that PTEB‐F‐10 possessed a more compact assembly, which might be due to the remained fluorosurfactants as evidenced from X‐Ray photoelectron spectroscopy (XPS) (Figure S6, Supporting Information). Selected area electron diffraction (SAED) revealed the amorphous nature of PTEB aerogels like many other CMPs,^26^, ^27^ which was attributed to the random 3D polymer network formed by rotatable diacetylene bonds. Interestingly, we found that fluorine content decreased with increasing initial fluorosurfactant concentration. This might be due to the high initial fluorosurfactant concentration result in a lower polymerization degree and a less perfect polymer network, thus the sucked surfactants in the gel matrix can be easier washed off during the purification process.

The large pores, such as mesopores and macropores are capable of enhancing the mass transfer, which is important for many energy‐related and adsorption applications. However, these pores are always negligible in CMPs, although a few works demonstrate their existence due to side reactions.^28^ Herein fluorosurfactants were used as an effective way to modulate the pore size distribution (PSD) by altering the crosslinking degree.^19^ As revealed by nitrogen adsorption/desorption test, both nitrogen isothermal curves of aerogels with/without using fluorosurfactants featured a combination of type I and type IV isothermal profiles (**Figure** **3**a).^29^, ^30^ However, the PTEB‐F‐10 showed a lower adsorption at low P/P_0_, while a sharper increase of adsorption at high P/P_0_, suggesting a much higher macro‐ and mesopores proportion than PTEB‐0. This conclusion can also be drawn from PSD diagrams derived from NLDFT method, where increase of pores larger than 2 nm was observed for PTEB‐F‐10 (Figure 3b, Figure S7, Supporting Information). The proportion of micropores was roughly estimated by comparing single‐point pore volume (V_micro_/V_tot_) calculated at p/p_0_ = 0.1 (for micropores) and p/p_0_ = 0.99 (for total pores), from which 68.6% and 31.4% was derived for PTEB‐0 and PTEB‐F‐10, respectively. This result was also in good agreement with calculation from cumulative PSD curves as shown in Figure S7, Supporting Information. All of above analysis point that fluorosurfactants can induce the formation of more large pores, important for efficient mass transfer. In this way, CMP aerogels can truly become a kind of hierarchical porous materials.

**Figure 3:**
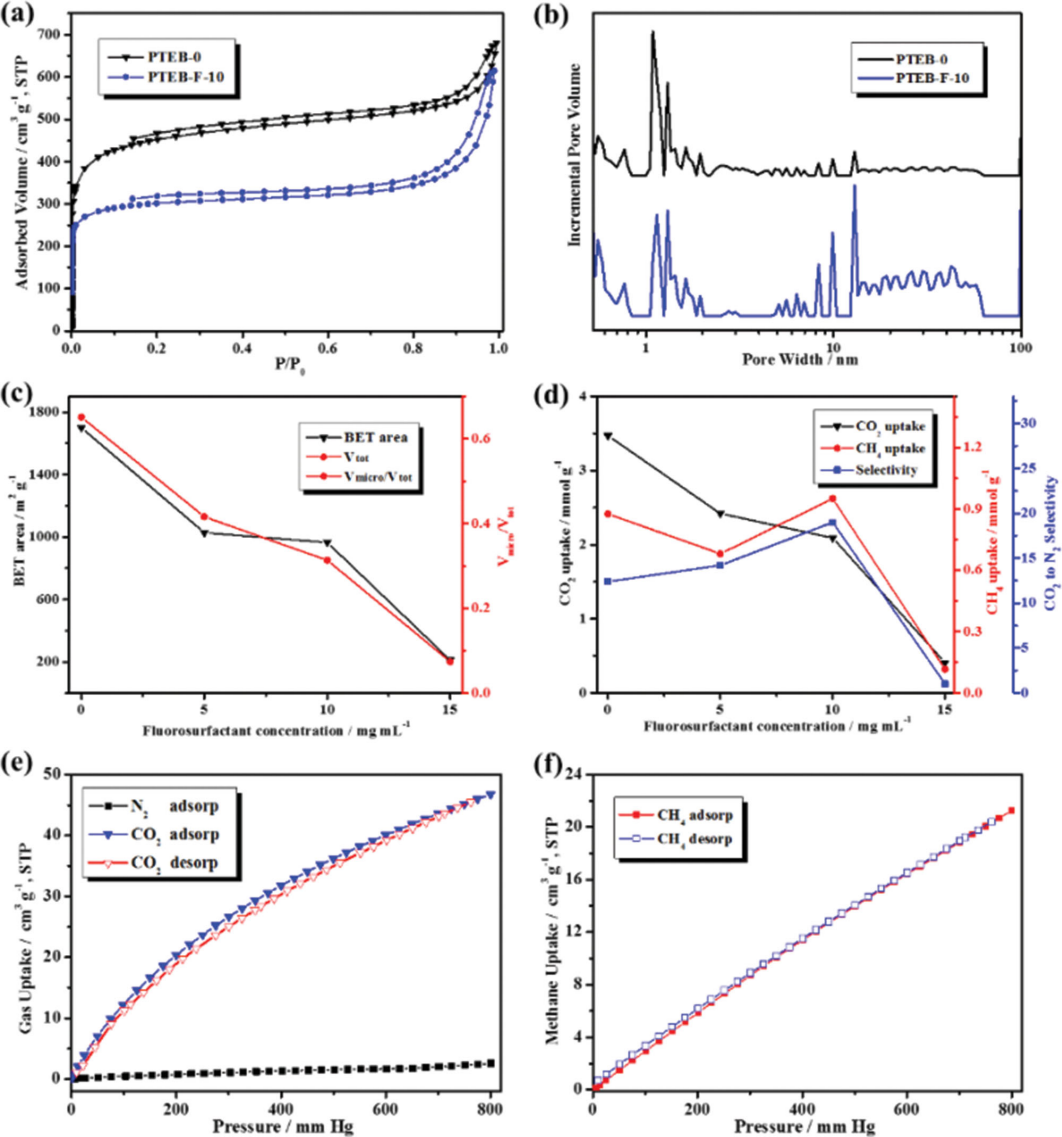
Gas sorption measurement. a, b) N_2_ sorption/desorption isothermals (77K) and pore size distribution (PSD, calculated by NLDFT method) of freeze‐dried PTEB‐0 and PTEB‐F‐10, respectively. c) Pore parameters vibration with increasing fluorosurfactant concentration. d) Gas sorption performance and CO_2_ to N_2_ selectivity (calculated by using adsorbed volume at 1 bar at 273 K) variation with increasing fluorosurfactant concentration. e) CO_2_ and N_2_ isothermal sorption curves of PTEB‐F‐10 at 273 K. f) CH_4_ isothermal adsorption/desorption curve of PTEB‐F‐10 at 273 K.

Every coin has two sides: the BET surface area of PTEB‐F‐10 was only 965 m^2^ g^−1^, much lower than that of PTEB‐0 although comparable to many conventional CMPs.^11^, ^28^, ^31^, ^32^ Two reasons can account for the decrease of BET area. First, a low polymerization degree results in less developed microporosity which was created by crosslinking of monomers. Therefore, the BET surface area decreases with the increased curvature radius of pores. There is a trade‐off relationship between BET surface area and large pores proportion. Second, remained fluorosurfactants may block the pores to some extent and thus decrease the specific surface area (SSA).

Systematical investigation of the effect of initial fluorosurfactant concentration has also been conducted. As shown in Figure 3c, both the BET surface area and micropores proportion were decreased with increasing fluorosurfactant concentrations, which was consistent with analysis described above. In this way, both the SSA and micropore proportion can be tuned in a wide range from 211 m^2^ g^−1^, 7.4% (PTEB‐F‐15) to 1701 m^2^ g^−1^, 65.1% (PTEB‐0). The tunable pore parameters facilitate manipulating the gas sorption performance (Figure 3d), where the adsorption capacity and selectivity can be controlled by using appropriate amount of surfactants.

Even though the BET surface area decreased with the addition of surfactants, the PTEB‐F‐10 still showed promising performance in gas sorption. As shown in Figure 3e, the aerogel showed moderate CO_2_ sorption capacity of 2.08 mmol g^−1^, among one of the excellent nitrogen‐ and metal‐free gas sorbents based on CMPs such as CMP‐0, CMP‐1, and TFM‐1 (typically around 2 mmol g^−1^).^13^, ^33^, ^34^ The CO_2_/N_2_ selectivity is also an important index to evaluate the practical applications of gas sorbents. The selectivity of PTEB‐F‐10 calculated by ideal adsorption solution theory (IAST) and initial slope method were 44.6 and 40.2, respectively, which was higher than or comparable to PTEB‐0 and a variety of porous materials including nitrogen‐doped microporous materials, nitrogen‐doped polyimine‐based carbons, imine‐linked porous polymer frameworks, and some microporous organic polymers.^35^, ^36^, ^37^, ^38^ Interestingly, the CH_4_ sorption capacity seems not positively correlate with BET surface area, as PTEB‐F‐10 showed even better performance than PTEB‐0 in this case (0.950 vs 0.875 mmol g^−1^) although with a much lower surface area (Figure 3f).^12^ This value was well within the range of best nitrogen‐ and metal‐free gas sorbents based on MOPs, including some high‐surface‐area COFs (0.36–0.67 mmol g^−1^), PAFs (0.80∼1.21 mmol g^−1^) and post‐metalated porous aromatic frameworks (less than 0.8 mmol g^−1^).^39^, ^40^, ^41^ The improvement of CH_4_ adsorption upon addition of the fluorosurfactant might be attributed to the increased pore volumes or the proportion of larger pores.

The reversibility of gas sorption is also highly preferred. As less external energy input will be required if the sorbents can be regenerated at mild conditions (e.g., at the room temperature), which was of paramount importance for industrial operation. Without strong interactions with adsorbates (i.e., physical sorption mechanism), adsorption/desorption curves of PTEB‐F‐10 for both CO_2_ and CH_4_ nearly overlapped (Figure 3c,d), suggesting fully reversible sorption can be achieved at near room temperature without much energy consume.

As well as a satisfactory gas sorption performance, the fluorosurfactant‐induced hierarchical porous structure and increase in hydrophobicity triggered the enhanced oil and organic solvent adsorption performance. The hierarchical porosity was proved by nitrogen adsorption test mentioned above, whereas the improvement of hydrophobicity was characterized by a light larger contact angle (ca. 7.5°) as shown in Figure S10, Supporting Information. As the integrity is essential for self‐supporting sorbents, supercritical‐drying was adopted to afford the aerogel with better mechanical properties (Figure S2, Supporting Information).

As shown in **Figure** **4**a, the PTEB‐F‐10 showed higher sorption capacity (20–48 times weight gain) towards a wide range of organic solvents and oils than that of fluorosurfactant‐free aerogels (10–31 times). The sorption performance of PTEB‐F‐10 was significantly higher than that of HCMP networks (1–16 times), porous covalent porphyrin framework (14.7–25.9 times) and even comparable to some macroporous graphene‐based carbon materials (<40 times).^42^, ^43^, ^44^ It should also be noted that the PTEB‐F‐10 exhibited an exceptional amine sorption capacity (17–25 times), much higher than that of efficient porphyrin‐integrated amine sorbents (<6 times).^45^ Highly hydrophobicity and hierarchical porosity endowed the aerogel with selective and fast oil sorption feature. In Figure 4b, the aerogel showed a discriminated sorption of acetone than the water. The aerogel can achieve 85% of its saturated sorption capacity within 10 seconds for acetone, while it showed no sorption of water with the time evolution. This high sorption capacity and fast kinetics might be attributed to the enhanced mass transfer from hierarchical porosity and improved hydrophobicity induced by fluorosurfactants. Owing to its high selectivity, the aerogel can also be directly applied in oil/water system, where the on‐water and under‐water sorption process can be accomplished within seconds (Figure 4c–d). More importantly, thanks to the high thermal stability of the aerogel, it can be simply regenerated by heating in the air to evaporate adsorbates after sorption. The sorption/regeneration cycles can be repeated without obvious sorption capacity loss (Figures S8,S9, Supporting Information).

**Figure 4:**
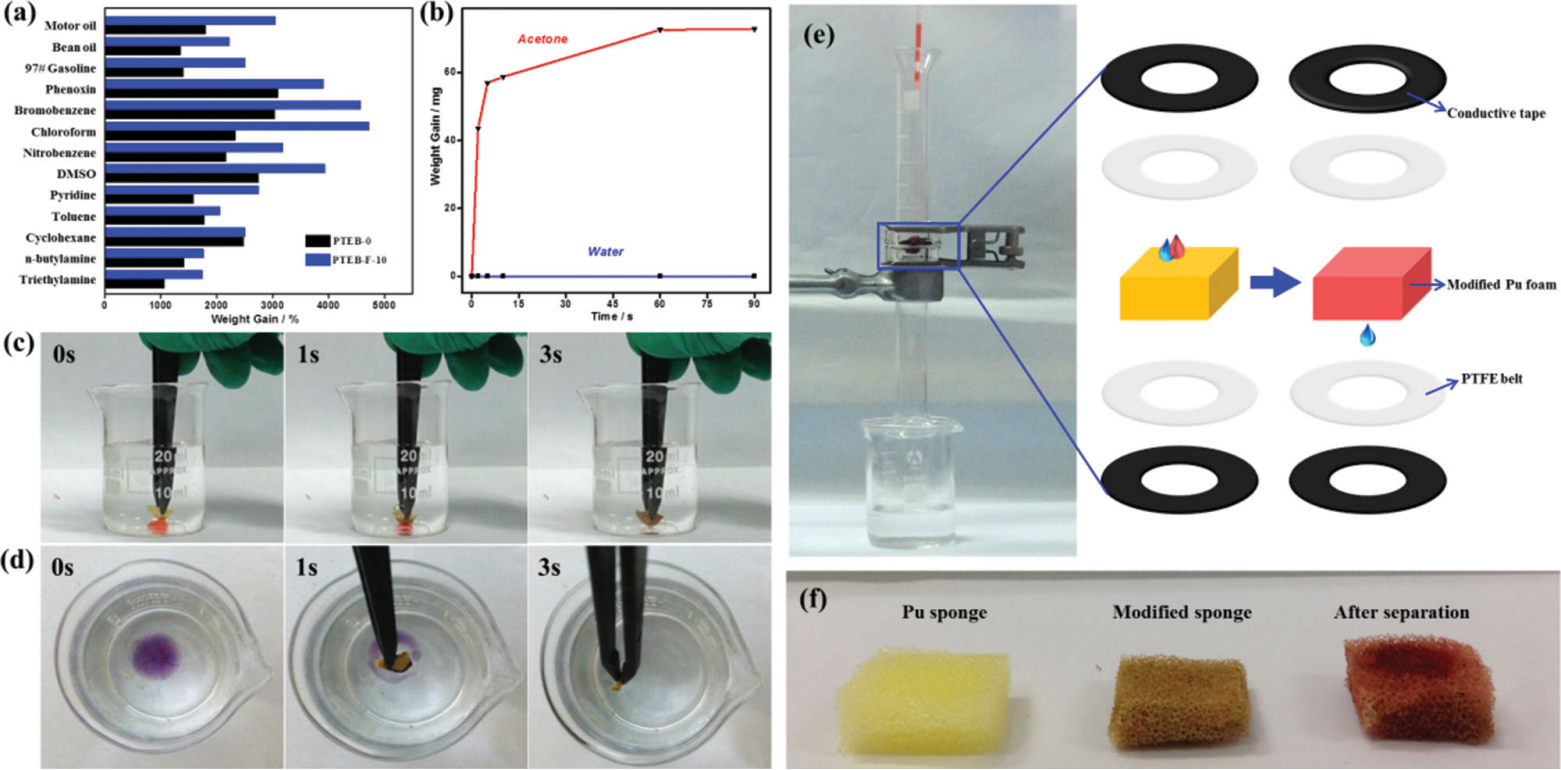
Oils sorption and oil/water separation test. a) The sorption capacity of PTEB‐0 (black bar) and PTEB‐F‐10 (blue bar) towards a variety of organic solvents and oils. b) Time‐dependent weight gain of PTEB‐F‐10 towards water (blue) and acetone (red). c,d) Underwater (chloroform, dyed with Sudan III) and on‐water sorption (toluene, dyed with Sudan Black B) of organic solvents by PTEB‐F‐10, respectively. e) Oil/water separation device and its work mechanism. f) Polyurethane sponges before modification, after aerogel‐modification and after separation process, respectively.

The high selectivity of the aerogel also inspired us to explore its oil/water separation ability. As shown in Figure 4e–f, Figures S11–S12, Supporting Information, the aerogel‐loaded polyurethane (Pu) sponge was assembled into a separation device. The mixture of water and dichloromethane (dyed by Sudan III) was used for the test. It can be seen that when the mixture solution was passed by, the modified Pu sponge can selectively block the organic phase and thus achieve oil/water separation. After separation, the sponge turned from brownish yellow to red, indicating the sorption of dyed solvent. The separation mechanism was based on the oil sorption of loaded aerogels. Since the Pu sponge consist of large pores about hundreds of micrometers (Figure S12, Supporting Information) and the surface was hydrophobic after modification, the water can directly pass through by gravity and cannot be adsorbed. The organic solvents and oils, however, would be adsorbed by the loaded aerogels decorated on the sponges during penetration. Based on this mechanism, the device can achieve a separation ratio higher than 95% for several water/oil systems before the sponge was saturated. Although this separation mechanism was restricted by the adsorption capacity of materials, whereas the saturated separator can be regenerated by removing adsorbed solvents (e.g., heating), allowing its recycling use.

In summary, we have developed a rational method, i.e., fluorosurfactant‐assisted Glaser coupling reaction to prepare homogeneous and hierarchical‐porosity CMP‐based aerogels with tunable pore structures and enhanced sorption performance. On the basis of this approach, fluorosurfactants were adopted to realize homogeneous dissolved oxygen concentration, controlled polymerization degree and chemical composition. In this way, homogeneous aerogels with hierarchical porosity and appropriate wettability are acquired. Benefiting from high specific surface area, fluorosurfactants‐induced hierarchical pores and enhanced hydrophobicity, the resultant aerogel not only showed a high gas sorption (CO_2_ and CH_4_) capacity in the front of CMP‐based materials, but also exhibited exceptional organic solvent and oil clean‐up performance outperformed most MOPs. This method may open a new era for handling an environmental‐atmosphere‐sensitive gelation system and preparing hierarchical porous materials for a wide range of environmental and energy‐related applications.

## Supporting information

As a service to our authors and readers, this journal provides supporting information supplied by the authors. Such materials are peer reviewed and may be re‐organized for online delivery, but are not copy‐edited or typeset. Technical support issues arising from supporting information (other than missing files) should be addressed to the authors.

Supplementary
