# Fluorosurfactants‐Directed Preparation of Homogeneous and Hierarchical‐Porosity CMP Aerogels for Gas Sorption and Oil Cleanup

**DOI:** 10.1002/advs.201500217

**Published:** 2015-09-25

**Authors:** Ran Du, Zhe Zheng, Nannan Mao, Na Zhang, Wenping Hu, Jin Zhang

*Adv. Sci.* **2015**, *2*, 1400006

The authors wish to correct Figure 3c of the original manuscript, in which the curve *V*_tot_ vs. fluorosurfactant concentration was inadvertently missed. Figure 3 is reproduced below with the corrected graph.

**Figure 3:**
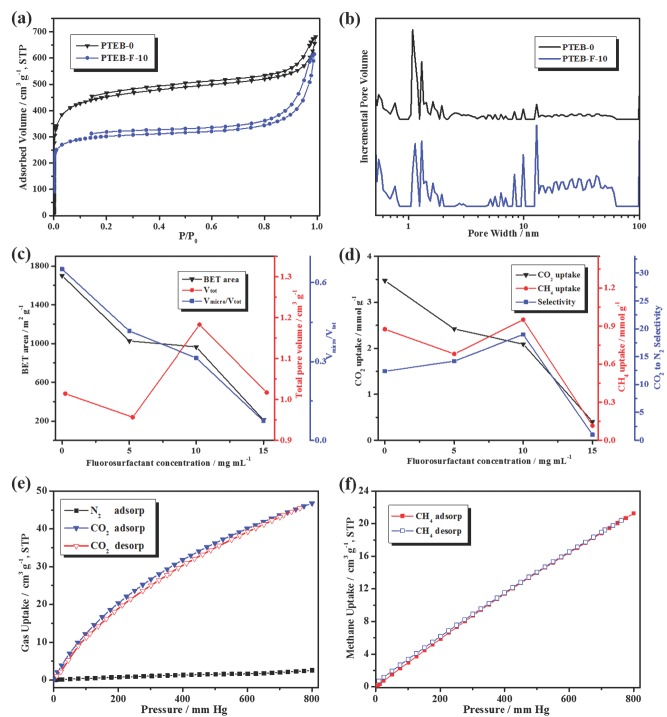
Gas sorption measurement. a, b) N_2_ sorption/desorption isothermals (77K) and pore size distribution (PSD, calculated by NLDFT method) of freeze‐dried PTEB‐0 and PTEB‐F‐10, respectively. c) Pore parameters vibration with increasing fluorosurfactant concentration. d) Gas sorption performance and CO_2_ to N_2_ selectivity (calculated by using adsorbed volume at 1 bar at 273 K) variation with increasing fluorosurfactant concentration. e) CO_2_ and N_2_ isothermal sorption curves of PTEB‐F‐10 at 273 K. f) CH_4_ isothermal adsorption/desorption curve of PTEB‐F‐10 at 273 K.

