# Supplementary material for: Fluorosurfactants‐Directed Preparation of Homogeneous and Hierarchical‐Porosity CMP Aerogels for Gas Sorption and Oil Cleanup
Source: Adv Sci (Weinh). 2015 Jan 28;2(1-2):1400006. doi: 10.1002/advs.201400006 (PMC5115277; doi:10.1002/advs.201400006)
Supplement: Supplementary file 1 — Supplementary [file ADVS-2-0a-s001.pdf]

Copyright WILEY-VCH Verlag GmbH & Co. KGaA, 69469 Weinheim, Germany, 2015.

Supporting Information

for *Adv. Sci.,* DOI: 10.1002/advs.201400006

Fluorosurfactants-Directed Preparation of Homogeneous and Hierarchical-Porosity CMP Aerogels for Gas Sorption and Oil Cleanup

Ran Du, Zhe Zheng, Nannan Mao, Na Zhang, Wenping Hu, and Jin Zhang**

Supporting Information for

Fluorosurfactants-Directed Preparation of Homogeneous and hierarchical-porosity CMP Aerogels for Gas Sorption and Oil Cleanup

Ran Du,[1]‡ Zhe Zheng,[1,2]‡ Nannan Mao[1], Na Zhang,[1] Wenping Hu[2] and Jin Zhang [1]**

R. Du, Z. Zheng, N. N. Mao, N. Zhang, Prof. J. Zhang
[1]Center for Nanochemistry, Beijing National Laboratory for Molecular Sciences, Key Laboratory for the Physics and Chemistry of Nanodevices, State Key Laboratory for Structural Chemistry of Unstable and Stable Species, College of Chemistry and Molecular Engineering, Peking University, Beijing 100871, P.R. China
E-mail: jinzhang@pku.edu.cn

Z. Zheng, Prof. W. P. Hu
[2]Collaborative Innovation Center of Chemical Science and Engineering (Tianjin), Department of Chemistry, School of Science, Tianjin University, Tianjin, 300072, P.R.China
E-mail: huwp@iccas.ac.cn

Part I Experiments

Reagents and materials

1,3,5-triethynylbenzene (TEB) was purchased from Alfa-Aesar Co.,Ltd., fluorosurfactant ($C_8F_{17}SO_2NHCH_2CH_2OH$) was obtained from Beijing FLUOBON Surfactant Institute. Other reagents were purchased from commercial suppliers (Alfa-Aesar, TCI or Aladdin Chemistry Co.,Ltd.). All reagents were used without further purification.

Preparation of poly(1,3,5-triethynylbenzene) (PTEB) Organogel

PTEB organogel was prepared by homocoupling of TEB with CuCl as catalyst in the pyridine solution as reported elsewhere.[1] Difference lies in the extra addition of specific amount of fluorosurfactant. In a typical formula, fluorosurfactant (20 mg, 0.037 mmol) and CuCl (5 mg, 0.05 mmol) were dispersed in pyridine (1 mL) in a glass bottle, followed by the addition of pyridine (1 mL) solution containing TEB (30mg, 0.2mmol). Then the mixture was subjected to sonication for ca. 10s, followed by grounding in the water bath at 40 $^{\circ}C$ for 72 h to allow the formation of PTEB organogel. The resultant gel was washed sequentially with pyridine, chloroform, methanol and ethanol, which ensure the complete removal of impurities.

Preparation of PTEB Aerogel

Two drying methods were employed to prepare aerogel. The former is freeze-drying, where the solvent inside PTEB wet gel was exchanged with water by the method similar to the purification step described above. Then the gel was flash-frozen by liquid nitrogen, followed by freeze-drying for 24 h. The latter method was supercritical fluid drying using CO_2 as medium. The solution inside PTEB wet gel was exchanged with alcohol, followed by subjecting in drying chamber of the Thar Supercritical Fluid Extraction. The ethanol solvent was replaced by liquid CO_2 by flushing the chamber for several minutes. The chamber was then sealed and heated above the critical point of CO_2 (31.1 $^{\circ}C$, 72.8 atm). After stabilizing for several minutes, the pressure in the chamber was slowly released over ca. 90 min at a constant temperature.

Part II Characterization

IR and Raman Spectroscopy

FT-IR spectra were recorded by micro FT-IR spectroscopy (Thermo Scientific NICOLET iN10 MX). The Raman spectra were obtained by Raman Imaging Microscope System (LabRAM HR800), with excitation laser wavelength at 632.8 nm.

Dissolved oxygen test

To test the dissolved oxygen (DO) evolution during the gelation process, a pen dissolved oxygen meter (AMT08) was used to record the real-time DO value.

To facilitate measurement and get a more clear picture, the reaction system was scaled up to 9 mL pyridine solution (contain 135mg TEB, 27mg CuCl, 0 or 135mg fluorosurfactant). The reaction took place in a glass bottom, where the total height of the solution was ~30 mm. For measurement, the pen was directly put into the reaction system and kept for 2~3 seconds before obtaining a DO value. The measurement position was ca. 1mm (top), 15mm (middle) and 29 mm (bottom) beneath the solution surface.

Firstly, the CuCl and fluorosurfactant were dissolved in 9 mL pyridine solution, and the first set of DO values (top, middle and bottom section) before reaction was obtained (the time was 0 min). Secondly, the TEB was added followed by mixing with assistance of sonication for ~15s, achieving a homogeneous solution. After then, a series set of DO values at different height position were recorded by a fixed time interval. The measurement was continued for ca. 5 hours until the gel appeared.

Electron Microscopy

Scanning electron microscopy (SEM) was performed on a Hitachi S-4800 field-emission-gun scanning electron microscope. The samples were prepared by directly putting aerogels on the conductive tape followed by gold sputtering.

Transmission electron microscopy (HRTEM) analysis was carried out by Tecnai F20 at 200 kV. The samples were prepared by dispersing aerogels in chloroform with assistance of ultrasonication, followed by dropping the dispersion onto carbon coated copper grids and drying at ambient temperature.

Element Analysis

X-Ray photoelectron spectroscopy (XPS) was performed using an Axis Ultra spectrometer (Kratos, UK) with a high-performance Al monochromatic source operated at 15 kV. The XPS spectra were taken after all binding energies were referenced to the C 1s neutral carbon peak at 284.8 eV, and the elemental compositions were determined from peak area ratios after correction for the sensitivity factor for each element. Deconvolution of the XPS C 1s peak was performed by CasaXPS.

Elemental analysis of C and H was further determined by using elemental analyzer (vario EL, from Elementar Analysensysteme GmbH).

Nitrogen Adsorption Measurement

Nitrogen adsorption experiments were performed with ASAP 2020 (Micromeritics, USA) at 77 K. The sample was outgassed at 45 °C for at least 15 hours under vacuum before measurement.

Thermal Properties Characterization

Thermogravimetric analysis (TGA) was conducted by Q600 STD with heating rate of 10 K min^{-1} in nitrogen or air atmosphere.

Contact Angle Measurement

Contact angle measurement was directly conducted on the aerogel at room temperature. The data was collected by OCA20 (Dataphysics).

Oil Uptake Measurement

Typically, a piece of aerogel (ca. 2 mg) was dropped into organic solvent or oil for 24 hours to achieve adsorption equilibrium. Then the weight gain (%) of the aerogel was calculated by

$$q_m=100*(m-m_0)/m_0$$

Where m_0 and m represent the mass of aerogel before and after adsorption, respectively.

The regeneration of aerogel was achieved by heating the saturated gel at 60 °C for a period of time (which was dependent on the adsorbed solvent, e.g. 10 min for acetone).

Oil/water Separation

The PTEB-F-10 was loaded on polyurethane (Pu) sponge by dipping the Pu sponge (with pore size of hundreds of micrometers) into the chloroform dispersion of PTEB-F-10 for 3 days. Then the modified Pu sponge was taken out and the remained solvent was evaporated by vacuum drying at 45 °C. The separation device was constructed by sandwiching the modified Pu sponge between two hollow glass-made cylinders with a clamp as shown in **Figure S11**. The oil/water mixture was prepared by mixing deionized water and dichloromethane (dyed by Sudan III) together.

For measurement, the oil/water mixture was dropped on the separator by a straw. When the mixed solution went through the modified sponge, the water was passed by while the dichloromethane was adsorbed, thus achieving the separation.

For a rough estimation of separation ratio for water/organic solvent system, following method was used.

After dropping the mixture of water and organic solvent (toluene, dichloromethane or chloroform), the mixed solution passing through (ca. 10 mL) the modified Pu foam was collected by a graduated cylinder (25 mL). Hence the solution volume (V_{tot}) can be directly read from the cylinder, and the weight of solution (m_{tot}) was obtained by weighing incremental mass of cylinder after collecting the solution. Hence the apparent density (ρ_{app}) of filtered solution can be calculated by

$$\rho_{app} = m_{tot} / V_{tot}$$

Since the water and organic solvent were immiscible, then we have

$$m_{tot} = \rho_w * V_w + \rho_{org} * V_{org},$$

$$V_{tot} = V_w + V_{org}$$

Then we can get that

$$V_{org} = (\rho_{app} - \rho_w) * V_{tot} / (\rho_{org} - \rho_w)$$

Then the separation ratio can be calculated as,

$$x = 1 - V_{org} / V_{tot} = (\rho_{org} - \rho_{app}) / (\rho_{org} - \rho_w)$$

Part III Figures

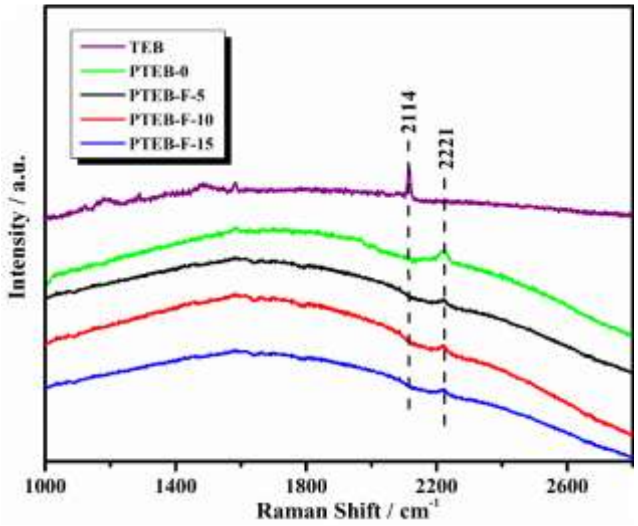

Figure S1. The Raman spectra of TEB monomer, PTEB-0, PTEB-F-5, PTEB-F-10 and PTEB-F-15.

The peak at 2114 cm^{-1} represented terminal C≡C stretching vibration, while the peak at 2221 cm^{-1} represented C≡C stretching vibration in phenyl diacetylene.[1-2] All PTEB aerogel with different initial fluorosurfactant concentration only showed the peak at 2221 cm^{-1}, suggesting successful coupling in all cases.

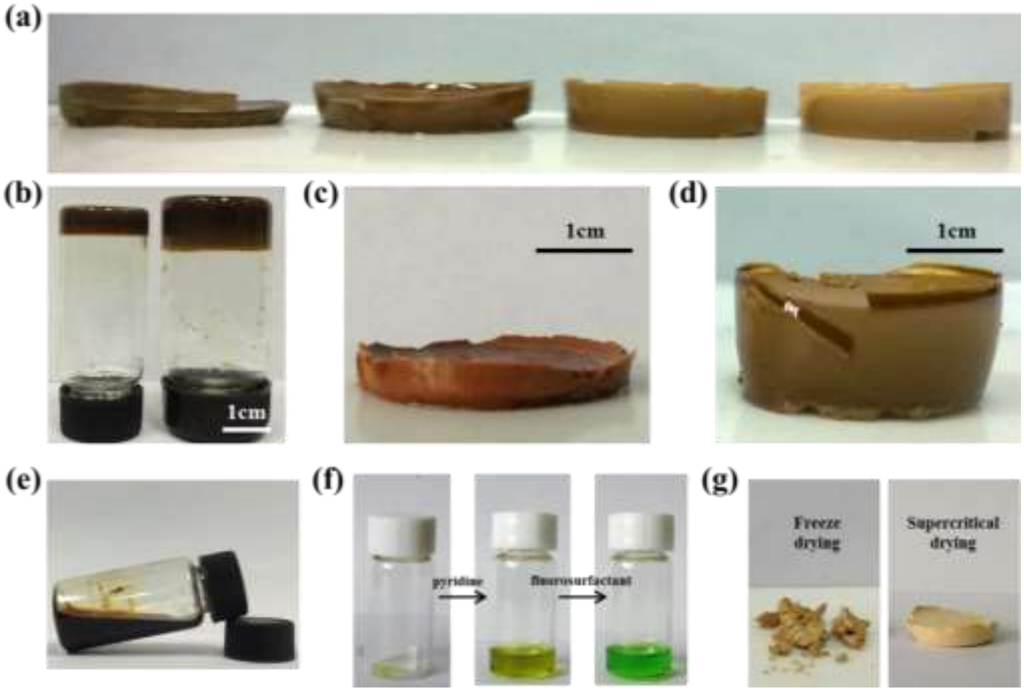

Figure S2. The digital graphs of a) wet gel derived from the same precursor solution volume but the different initial fluorosurfactant concentration of 0, 5, 10 and 15 mg mL^{-1}, from left to right. b) PTEB organogel synthesized with initial fluorosurfactant concentration of 0 and 15mg mL^{-1}, respectively. c) and d) The corresponding free-standing wet gels images of b). e) The product with initial fluorosurfactant concentration of 30 mg mL^{-1}. f) The evolution of pyridine solution of CuCl upon addition of fluorosurfactant. g) The aerogels obtained from freeze-drying and supercritical drying.

In S2a, the gradually lighter color of the gel suggested the gradually lower polymerization degree with increasing initial fluorosurfactant concentration. The slightly increased gel height from left to right indicate that for surfactant-free gel, only a part of system (top section) can form the gel, limiting its scaling production.

S2b-d indicated that addition of enough fluorosurfactant can result into homogeneous and “big” gel, important for scaling production.

S2e indicated that too much fluorosurfactant would suppress the gelation process.

S2f showed that the color of pyridine solution turned from olive to green upon the addition of fluorosurfactant, suggesting the coordination of Cu(I).

S2g showed that supercritical drying often resulted into integral and crack-free aerogels with better mechanical strength than freeze-dried samples.

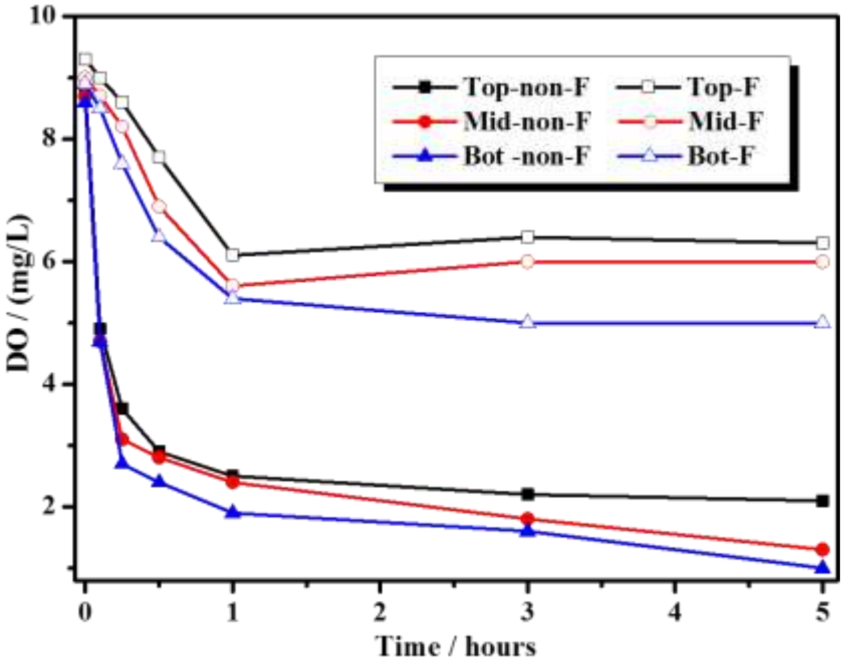

Figure S3. The in-situ dissolved oxygen (DO) measurement at different solution height in the reaction system with (the hollow data point) and without (the solid data point), respectively. The inspection time was end at 5 hours when the gel occurred.

Both reaction system (with or without addition of fluorosurfactants) underwent obvious oxygen consumption at initial stage (about 1 hour). However, it can be seen that fluorosurfactants-containing system can significantly suppress the oxygen consumption compared with surfactant-free system.

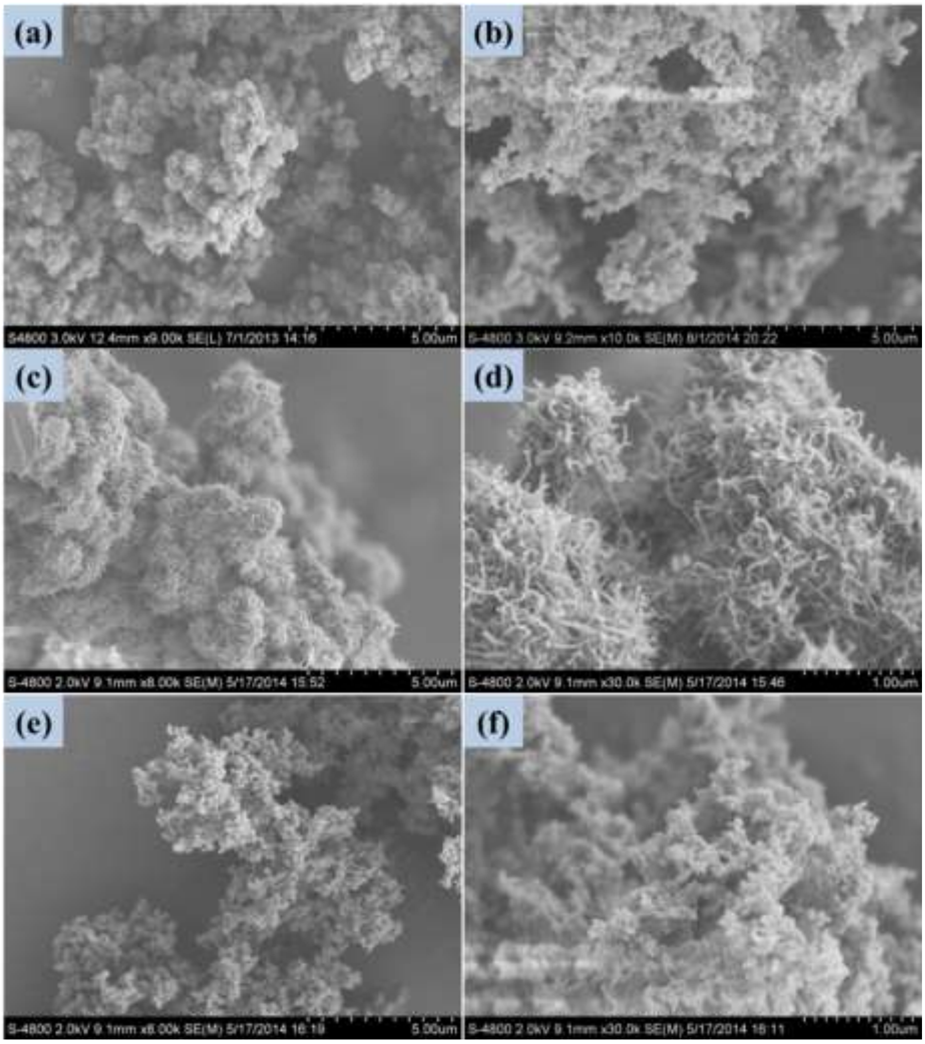

Figure S4. SEM images of a) PTEB-0 and b) PTEB-F-10 obtained by freeze-drying. SEM images of c) & d) PTEB-0 and e) & f) PTEB-F-10 obtained by supercritical CO_2 drying.

The PTEB-0 was composed of short fiber-like building blocks, while PTEB-F-10 was constructed with nanoparticles. The drying method seems do not have much effect on the aerogel morphology.

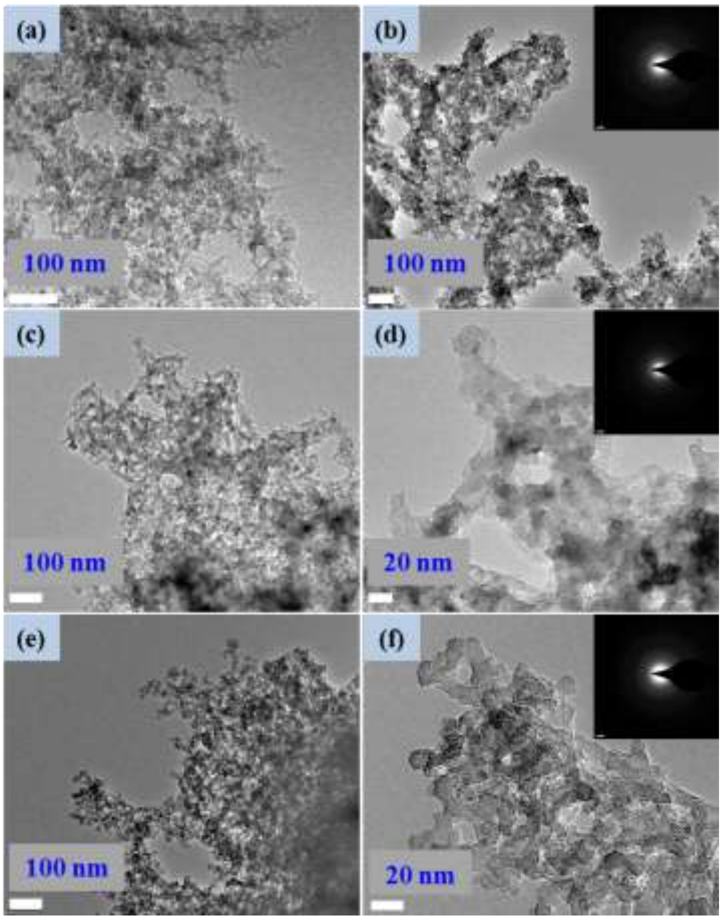

Figure S5. TEM images of a) PTEB-0 and b) PTEB-F-10 obtained by freeze-drying. TEM images of c) & d) PTEB-0 and e) & f) PTEB-F-10 obtained by supercritical CO_2 drying.

The structure of PTEB-0 looks like more loosely than that of PTEB-F-10. The drying method seems do not have much effect on the aerogel morphology.

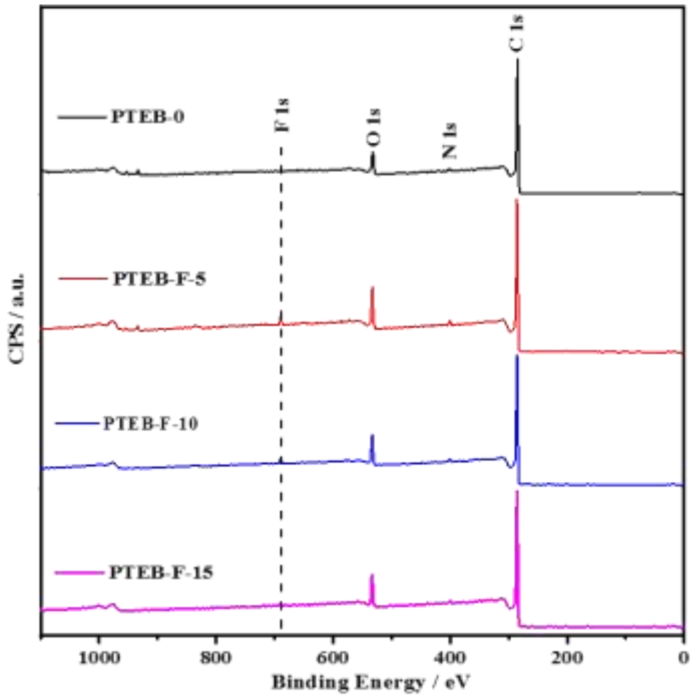

Figure S6. The full range XPS spectra of PTEB aerogels synthesized without and with fluorosurfactant.

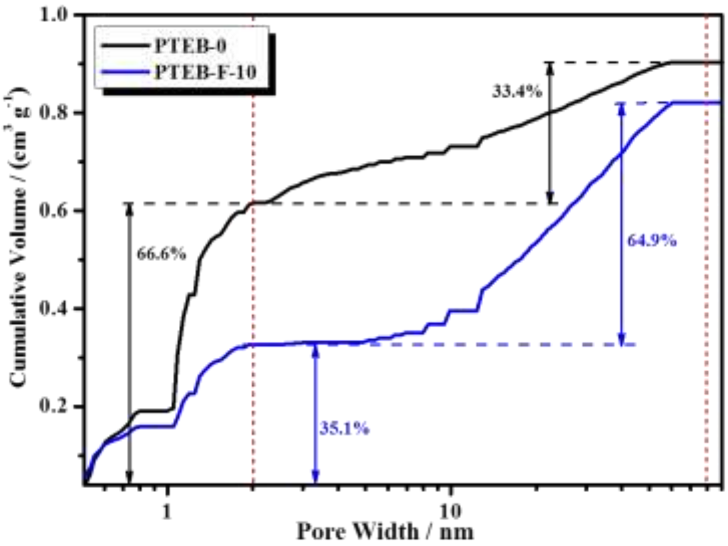

Figure S7. The cumulative volume curves of freeze-dried PTEB-0 and PTEB-F-10.

It showed that PTEB-0 possessed 66.6 % micropores (with pore width less than 2 nm), while PTEB-F-10 possessed only 35.1 % micropores, suggesting the role of fluorosurfactant on increasing the ratio of larger pores.

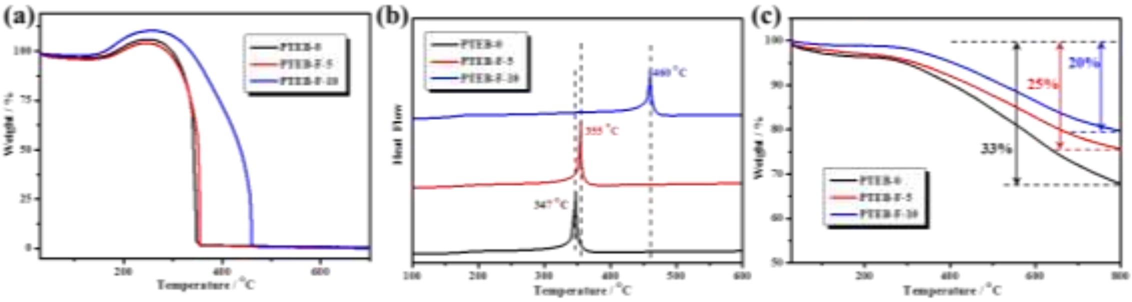

Figure S8. a) TGA and b) DSC curves of PTEB aerogels with different initial fluorosurfactant concentration in the air atmosphere. c) showed the TGA curves of them in the nitrogen atmosphere.

The addition of fluorosurfactants can improve the thermal stability of the PTEB aerogel to some extent. This can be evidenced from both the positive shift of exothermal peak (from 347 to 460 °C) of DSC curves and less weight loss (from 33 to 20 %) of TG curves, with the increase of initial fluorosurfactant concentration.

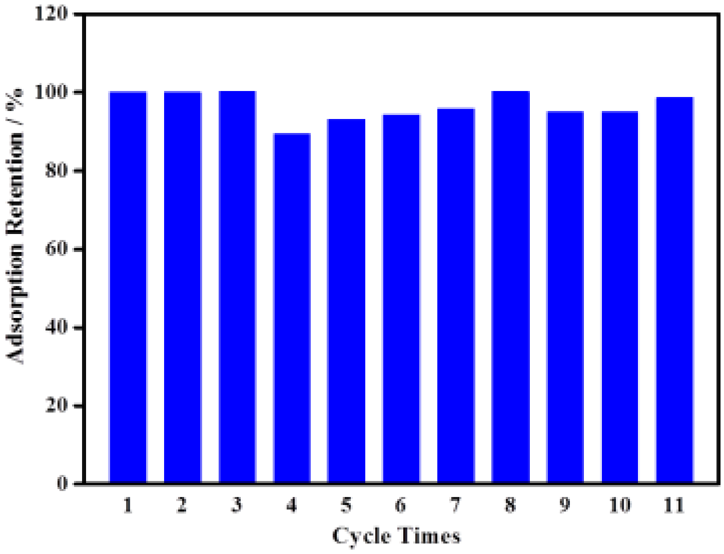

Figure S9. Adsorption capacity retention of the PTEB-F-10 towards acetone by heating regeneration at 60 °C for 11 cycles.

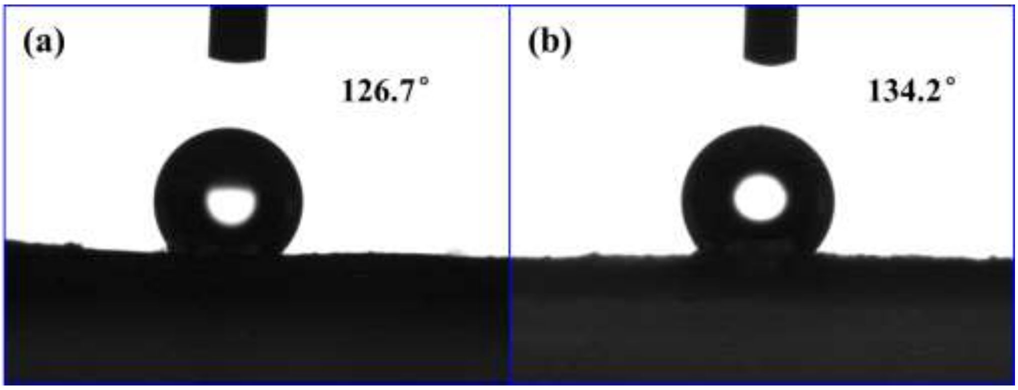

Figure S10. The contact angle of a) PTEB-0 and b) PTEB-F-10.

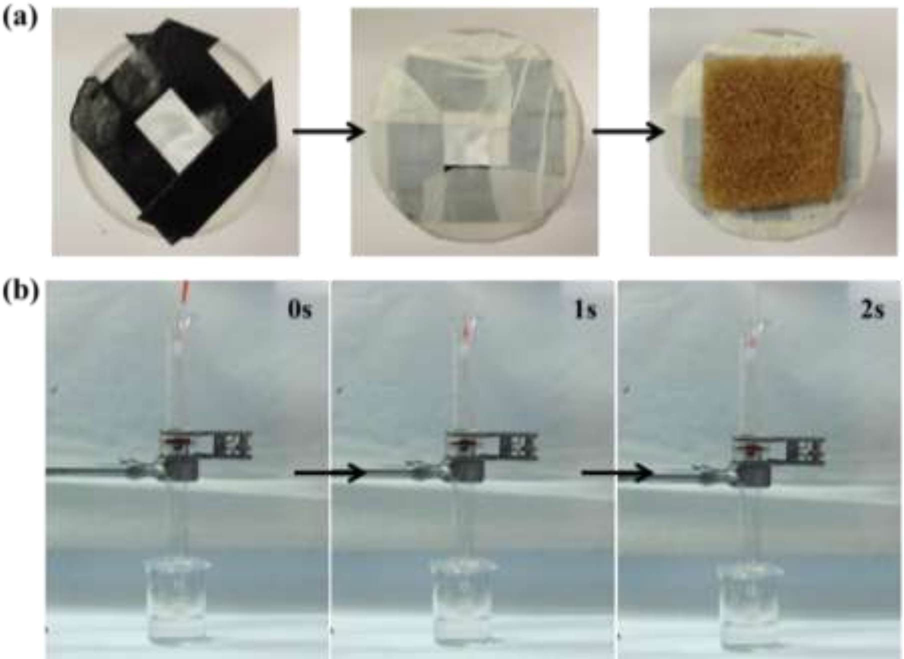

Figure S11. a) The assembly procedure of the oil/water separation device. Stick the conductive tape on the glass-made hollow cylinder, stick the poly(tetrafluoroethylene) (PTFE) belt on the conductive tape, and then put the modified Pu sponge on the top of it. Finally, another cylinder (with conductive tape and PTFE belt sticking on it) was coated on it and fixed by a clamp. b) The demonstration of separation process by dropping the mixture of water and dichloromethane (dyed by Sudan III) in the device.

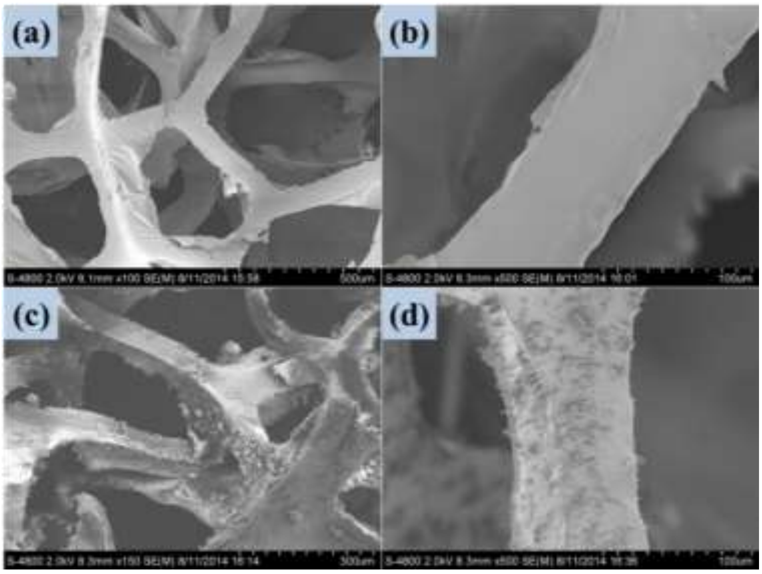

Figure S12. SEM images of polyurethane sponge a) & b) before and after c) & d) loading with aerogels.

Part IV Tables

Table S1. Summary of elemental atomic percentage obtained by XPS of different kinds of PTEB aerogels.

Element	PTEB	PTEB-F-5	PTEB-F-10	PTEB-F-15
C	91.74	86.86	89.32	89.73
O	7.57	9.80	7.88	8.53
N	0.52	1.49	1.52	1.05
F	0	1.37	1.04	0.48
S	0	0.11	0.10	0.09
Cu	0.18	0.24	0.06	0.04
Cl	0	0.14	0.09	0.09

Table S2. Summary of porosity and nitrogen adsorption data of PTEB aerogels derived from different reaction conditions.

Sample	$V_{0.1}$[a] / $cm^3\ g^{-1}$	V_{total}[b] / $cm^3\ g^{-1}$	$V_{0.1}/V_{total}$	S_{BET}[c] / $m^2\ g^{-1}$
PTEB-0	0.6608	1.0144	0.6514	1701
PTEB-F-5	0.3981	0.9568	0.4161	1027
PTEB-F-10	0.3711	1.183	0.3137	965
PTEB-F-15	0.0756	1.017	0.0743	211

[a] **Pore volume derived at p/p_0=0.1.**

[b] **Pore volume derived at p/p_0=0.99.**

[c] **BET surface area was calculated in the partial pressure (p/p_0) range of 0.01–0.1 to give the best linearity.**

[d] **Typical condition: With initial monomer concentration c_0=15 mg mL^{-1}, catalysts (CuCl) amount of 16 wt%, reaction temperature of 40 ^{O}C, freeze-dried with water.**

Table S3. Summary of CO_2 and CH_4 adsorption data of various materials.

Materials	BET surface area / m^2 g^{-1}	CO_2 adsorption / mmol g^{-1}	CH_4 adsorption / mmol g^{-1}	Metals or nitrogen containing	Reference
PTEB aerogel	965~1701	0.41~3.47 [a]	0.12~0.95	/	**This work**
Different functionalized CMP-1	522~1043	1.60~2.05 [a]	/	CMP-1-(NH_2) contains nitrogen	3
CMP-0	1018	2.10 [a]	/	/	4
TFM-1	738	1.73 [a]	/	Contain nitrogen	5
PPN-6, PPN-6-SO3H, and PPN-6-SO3Li	1186~4023	1.1~3.1 [b]	/	Contain lithium	6
Hypercrosslinked polythiophene and polypyrrole	437~726	2.7~2.9 [a]	/	Contain nitrogen	7
COP-1, COP-2	158~168	0.9~1.5 [a]	/	Contain nitrogen	8
BILP-3, BILP-6	1261~1306	5.12 [a]	/	Contain nitrogen	9
PGF-1, PGF-2	700~825	1.6~1.8 [a]	/	/	10
POPs	485~630	1.4~2 [a]	/	/	11
PIMs	/	1.5~2.6 [a]	/	/	12
PSN-1, PSN-2	376~1045	3.4 [a]	/	Contain nitrogen	13
COF-10, COF-102	1760~3620	1.34~1.79 [a]	0.36~0.67 [a]	/	14
PAF	2246~3857	2.07~3.48 [a]	0.80~1.21 [a]	/	15
PECONF	449~851	1.86~3.49 [a]	0.83~1.07 [a]	Contain nitrogen	16
PAF-26-COOM	430~717	2.32~2.81 [a]	0.67~0.80 [a]	Contain Li, Na, K or Mg	17

[a] Data collected at 273 K, 1 bar.

[b] Data collected at 295 K, 1 bar.

Table S4. Summary of oil uptake capacity of various adsorbents.

**Absorbent**	**Oil**	**Sorption capacity g g^{-1}**	**Reference**
PTEB aerogel	Amines, DMSO, chloroform, nitrobenzene, phenoxin, toluene, pyridine, pump oil, motor oil, etc.	20~48	**This work**
Porous covalent porphyrin framework	n-pentane, n-hexane, n-octane, gasoline, etc.	14.7~25.9	18
HCMP	Kerosene, ethyl acetate, dichloromethane, nitrobenzene, toluene, etc.	1~16	19
HCMP-1 and HCMP-2	Hexane, DMF, ethanol, nitrobenzene, chloroform, toluene, pump oil, etc.	6~33	20
MP1–CMP, ZnP2–CMP	Amine liquid	<6	21
Nanoporous PDVB	n-heptane, benzene	8~16	22
Hyper-crosslinked polymer nanospheres	n-hexane, toluene	0.5~2.5	23
Polyurethane based sponge	Octane, decane, dodecane, lubricating oil	13~20	24
Nanowire membrane	oils and some organic solvents	4–20	25
Activated carbon	benzene, toluene	<1	26
Graphene/a-FeOOH composite	cyclohexane, toluene, vegetable oil, etc.	10-30	27
Reduced graphite oxide foam	cyclohexane, chlorobenzene, toluene, petroleum, motor oil	5-40	28

References

[1] R. Du, N. Zhang, H. Xu, N. N. Mao, W. J. Duan, J. Y. Wang, Q. C. Zhao, Z. F. Liu, J. Zhang, *Adv. Mater.* **2014**, DOI: 10.1002/adma.201403058.

[2] M. J. Schultz, X. Zhang, S. Unarunotai, D.-Y. Khang, Q. Cao, C. Wang, C. Lei, S. MacLaren, J. A. Soares, I. Petrov, *Proc. Natl. Acad. Sci. U. S. A.* **2008**, *105*, 7353.

[3] R. Dawson, D. J. Adams, A. I. Cooper, *Chem. Sci.* **2011**, 2, 1173.

[4] S. Ren, R. Dawson, A. Laybourn, J.-x. Jiang, Y. Khimyak, D. J. Adams, A. I. Cooper, *Polym. Chem.* **2012**, 3, 928.

[5] X. Zhu, C. Tian, S. M. Mahurin, S.-H. Chai, C. Wang, S. Brown, G. M. Veith, H. Luo, H. Liu, S. Dai, *J. Am. Chem. Soc.* **2012**, 134, 10478.

[6] W. Lu, D. Yuan, J. Sculley, D. Zhao, R. Krishna, H.-C. Zhou, *J. Am. Chem. Soc.* **2011**, 133, 18126.

[7] Y. Luo, B. Li, W. Wang, K. Wu, B. Tan, *Adv. Mater.* **2012**, 24, 5703.

[8] H. A. Patel, F. Karadas, A. Canlier, J. Park, E. Deniz, Y. Jung, M. Atilhan, C. T. Yavuz, *J. Mater. Chem.* **2012**, 22, 8431.

[9] M. G. Rabbani, T. E. Reich, R. M. Kassab, K. T. Jackson, H. M. El-Kaderi, *Chem. Commun.* **2012**, 48, 1141.

[10] R. Kumar, V. M. Suresh, T. K. Maji, C. Rao, *Chem. Commun.* **2014**, 50, 2015.

[11] V. Guillerm, Ł. J. Weseliński, M. Alkordi, M. I. H. Mohideen, Y. Belmabkhout, A. J. Cairns, M. Eddaoudi, *Chem. Commun.* **2014**, 50, 1937.

[12] C. R. Mason, L. Maynard-Atem, K. W. Heard, B. Satilmis, P. M. Budd, K. Friess, M. Lanč, P. Bernardo, G. Clarizia, J. C. Jansen, *Macromolecules* **2014**, 47, 1021.

[13] G. Li, B. Zhang, J. Yan, Z. Wang, *Chem. Commun.* **2014**, 50, 1897.

[14] H. Furukawa, O. M. Yaghi, *J. Am. Chem. Soc.* **2009**, 131, 8875.

[15] T. Ben, C. Pei, D. Zhang, J. Xu, F. Deng, X. Jing, S. Qiu, *Energy Environ. Sci.* **2011**, 4, 3991.

[16] P. Mohanty, L. D. Kull, K. Landskron, *Nat. Commun.* **2011**, 2, 401.

[17] H. Ma, H. Ren, X. Zou, S. Meng, F. Sun, G. Zhu, Polym. Chem. **2014**, 5, 144.

[18] X.-S. Wang, J. Liu, J. M. Bonefont, D.-Q. Yuan, P. K. Thallapally, S. Ma, *Chem. Commun.* **2013**, 49, 1533.

[19] D. Tan, W. Fan, W. Xiong, H. Sun, A. Li, W. Deng, C. Meng, *Eur. Polym. J.* **2012**, 48, 705.

[20] A. Li, H.-X. Sun, D.-Z. Tan, W.-J. Fan, S.-H. Wen, X.-J. Qing, G.-X. Li, S.-Y. Li, W.-Q. Deng, *Energy Environ. Sci.* **2011**, 4, 2062.

[21] X. Liu, Y. Xu, Z. Guo, A. Nagai, D. Jiang, *Chem. Commun.* **2013**, 49, 3233.

[22] Y. Zhang, S. Wei, F. Liu, Y. Du, S. Liu, Y. Ji, T. Yokoi, T. Tatsumi, F.-S. Xiao, *Nano Today* **2009**, 4, 135.

[23] F. S. Macintyre, D. C. Sherrington, L. Tetley, *Macromolecules* **2006**, 39, 5381.

[24] Q. Zhu, Q. Pan, F. Liu, *J. Phys. Chem. C* **2011**, 115, 17464.

[25] J. Yuan, X. Liu, O. Akbulut, J. Hu, S. L. Suib, J. Kong, F. Stellacci, *Nat. Nanotech.* **2008**, 3, 332.

[26] M. Lillo-Ródenas, D. Cazorla-Amorós, A. Linares-Solano, *Carbon* **2005**, 43, 1758.

[27] H.-P. Cong, X.-C. Ren, P. Wang, S.-H. Yu, *ACS nano* **2012**, 6, 2693.

[28] Z. Niu, J. Chen, H. H. Hng, J. Ma, X. Chen, *Adv. Mater.* **2012**, 24, 4144.
